# Academic Stress and Sleep Quality among Chinese Adolescents: Chain Mediating Effects of Anxiety and School Burnout

**DOI:** 10.3390/ijerph20032219

**Published:** 2023-01-26

**Authors:** Hua Wang, Xiaoyan Fan

**Affiliations:** 1 School of Sociology and Population Studies, Nanjing University of Posts and Telecommunications, Nanjing 210023, China; 2 School of Social Development, East China Normal University, Shanghai 200241, China

**Keywords:** academic stress, sleep quality, anxiety, school burnout, Chinese adolescents

## Abstract

Previous studies have investigated the associations between academic stress and adolescents’ psycho-social development. However, the direct and indirect affecting mechanism of academic stress on sleep quality among Chinese adolescents have rarely been investigated. Using a self-report questionnaire data of 1232 adolescents in Jiangsu province, the purpose of the study is to investigate the relationships among academic stress, anxiety, school burnout, and sleep quality. Structural equation modeling (SEM) with Amos 25.0 was used to test the model fit in the present study. The results revealed that academic stress tends to have significantly direct effects on adolescents’ sleep quality. Moreover, anxiety and school burnout could mediate the associations between academic stress and sleep quality absolutely and sequentially. This study reveals the mechanism of the effect of academic stress on adolescents’ sleep quality and also develops the chain mediating model of anxiety and school burnout. In addition, the present study may shed light on social policy and social work intervention toward adolescents’ sleep quality.

## 1. Introduction

The healthy growth and development of adolescents is of great importance to both families and the nation. For the Chinese youth, education is not only an important way to support national prosperity, but also can lay the foundation for individuals’ future development. According to the data of the Ministry of Education in 2021, the number of students enrolled in compulsory education is 34.88 million, whereas that of general undergraduate students in higher education is 4.45 million [1]. This means that students are faced with fierce competition to have a chance to enter higher education [2]. As a result, they have to spend more time to complete heavy academic tasks, which puts them under considerable academic stress [3]. Considered as a major source of pressure for adolescents, academic stress mainly involves lack of confidence in expected school tasks and fear of academic failure [4]. After prolonged exposure to high academic stress, adolescents may experience anxiety, depression, and sleep disorders [5]. According to a Chinese report in 2018, adolescents with sleep quality problems is in a high rate of 76.01%. Given the high amount of homework and pressure to advance to higher education, the higher grades teenagers have poorer sleep quality [6].

Notably, some studies have investigated the affecting path between academic stress and sleep quality, and found that school burnout and anxiety may play a mediating role in the associations, respectively. For example, a study of secondary school students in Fuzhou, China, found that academic stress was not only directly related to sleep quality, but also exerted an influence through the mediating effect of school burnout [7]. Some relevant researchers found that anxiety played a mediating role between perceived stress and sleep quality in nurses, so these findings suggest that reducing perceived stress can help reduce the individuals’ anxiety symptoms and thus improve sleep quality [8]. However, the mediating effects of school burnout and anxiety have not been fully explored in adolescents in the Chinese context. The possible reason is that some scholars consider that the two variables may overlap, and it is not reasonable to analyze them using one framework [9]. In fact, burnout is the result of a positive emotional decline caused by chronic fatigue, whereas anxiety manifests as a state of mental abnormality under prolonged emotional exhaustion; thus, the two variables are subsequently related in a non-overlapping relationship [10]. In terms of the school environment, students may develop school burnout if they are chronically exposed to anxiety [11]. Therefore, to better understand the influencing mechanisms, we attempt to construct an analytical model to explore the mediating effects of anxiety and school burnout between academic stress and sleep quality. Thus, this study could provide guidelines for social work intervention of adolescents’ academic stress and its consequences.

## 2. Literature Review

### 2.1. The Effect of Academic Stress on Adolescents’ Sleep Quality

As one of the important indicators of quality of life, sleep quality is the individual’s overall satisfaction with his/her sleep experiences, which mainly includes sleep onset latency, sleep duration, sleep efficiency, and revitalizing feeling upon awakening [12]. The decline in sleep quality is influenced by external stress. For example, occupational stress affects the sleep quality of psychiatric nurses negatively, and the more stressful it is, the worse the sleep quality [13]. Elevated levels of stress in students are also significantly associated with low levels of sleep quality [14]. According to the stress process model, individuals’ behaviors are more likely influenced by social structures and pressures [15]. Chronic social stress can negatively affect individuals’ behaviors, which can cause an increased risk of sleep quality decline and insomnia. From this theoretical perspective, there is a strong correlation between academic stress and sleep quality among adolescents who are studying. Specifically, academic stress increases adolescents’ role tension and weakens their self-awareness, which in turn decreases their sleep quality. For example, data from the American College Health Association’ survey show that a major and growing problem is that students’ sleep quality decreases with increasing levels of academic stress [16]. Another cross-sectional study of Saudi Arabia also shows that academic stress contributes to poor sleep quality among students and this risk is four times higher than for students without stress [17].

Similarly, studies in a Chinese context have come to similar conclusions that adolescents’ academic stress is negatively associated with sleep quality [7]. This similarity suggests that at the high school level, adolescents commonly suffer from low sleep quality including difficulty falling asleep, insomnia, excessive dreaming, and inefficient sleep due to high academic stress. A middle school in south-central China also confirms this finding, suggesting that academic stress has a significant negative effect on sleep quality; the greater the academic stress, the poorer the sleep quality [18]. Although these previous studies have focused on the direct effect of academic stress on adolescents’ sleep quality, few studies have examined the mediating factors, especially for Chinese adolescents.

### 2.2. Anxiety as a Mediator

As the most common psychiatric symptom, anxiety is an abnormal state in which individuals experience negative psychological aspects, such as uneasiness, worry, or fear. This symptom includes both state anxiety, an acute response to stress, and trait anxiety, that is a chronic state expressing negative emotions; moreover, the negative effects of state anxiety eventually accumulate to trait anxiety [19]. However, regardless of type, anxiety is caused by external stress. To understand the relationship between stress and anxiety, researchers often use the biopsychosocial model [20] that suggests that anxiety is the result of a combination of biological, psychological, and social factors. Empirical studies have shown that social stress interacting with psychosocial resilience can cause physiological distress, which leads to anxiety [21]. In the case of adolescent anxiety, the academic stress, including leaving home to study in an unfamiliar environment, the amount and complexity of their studies, and the fear of poor performance, overwhelms their immature resilience capacity and leads to anxiety. For example, a cross-sectional study of Indian adolescents shows that academic competitive stress caused by socio-demographic factors has a significant positive association with anxiety [22]. Similarly, as a large educational country, China shows considerable academic stress among adolescents, exerting the most dominant and direct influence on anxiety among adolescents in mainland China [23]. Another explanatory theory focuses on adolescent identity perception and role orientation [24]. Adolescents’ misconceptions about their academic abilities lead to stereotypes of “not being able to learn”. In other words, many adolescents believe that “I am not a good learner” and perceive the learning content and process as academic stress, which leads to anxiety. These findings provide an empirical basis for research to reduce adolescents’ academic burdens in China. However, notably, a study of Massachusetts adolescents in the United States shows no significant relationship between adolescent stress and anxiety [25]. Thus, this association needs further validation.

Previous empirical studies have also shown that adolescent anxiety leads to reduced individual sleep quality and that those with anxiety commonly have low sleep quality [26]. For example, Austrian adolescents show that anxiety has a significant positive association with sleep quality. Moreover, among several physical and mental health parameters, anxiety is the strongest predictor of poor subjective sleep quality [27]. In southeastern China, academic stress among adolescents is not only directly related but also indirectly related to sleep quality through the mediating effect of anxiety [7]. Similarly, a cross-sectional study of 5226 adolescents in the central provinces of China show that high levels of anxiety are the most significant factor affecting their sleep quality [28]. This finding suggests that the emergence of anxiety among adolescents has a negative impact on their sleep quality, and thus, anxiety can be regarded as a mediator between academic stress and sleep quality.

### 2.3. School Burnout as a Mediator

Burnout is an individual response to negative emotional and interpersonal stress and includes three dimensions of exhaustion, disgust, and inefficiency [29]. Burnout is initially used only to understand chronic fatigue states in occupations. However, the similarity of school settings, where students have to study for long periods of time to pass exams, has led to the application of the burnout concept to adolescent populations as well. School burnout is a valid and uniquely important predictor of risk factors for delinquent behavior in adolescents [30]. School burnout is defined as a psychiatric response related to the stress of studying, which refers to chronic fatigue resulting from such excessive effort for a long period of time [30]. This symptom is manifested by the adolescent student’s powerlessness towards learning activities, contempt for the meaning of learning, and lack of achievement in academic outcomes.

Given that school burnout is associated with social stress, academic stress among adolescents has received special attention in literature. According to the transactional model, stress is a transactional relationship between individuals and their environment [31] and is controlled if the ability and resources to cope with the stress response change on this basis. Otherwise, stress may cause distress to the individual. In the case of adolescents, who are at a critical stage of learning, their coping abilities and available resources are relatively scarce when confronted with academic stress. Thus, their psychological and behavioral effects are adversely affected. Several empirical studies have shown that academic stress has a significant positive correlation with school burnout. Moreover, the greater the academic stress faced by adolescents as they grow older, the more severe is their school burnout [32]. Similar findings are also found in mainland China. For middle school students, academic stress is a risk factor for school burnout, and at high levels, greatly increases the likelihood of experiencing school burnout [33].

Admittedly, as a dysfunctional psychosomatic state, school burnout represents a multidimensional emotional response to an individual’s inability to cope with stress. Empirical studies have shown that school burnout often has negative consequences on adolescents’ daily behaviors, including sleep quality issues such as sleep efficiency, latency, and duration [34]. Among adolescents in southeast China, school burnout is negatively associated with sleep quality and also mediated an indirect negative relationship between academic stress and sleep quality [7]. For college students, the relationship between school burnout and sleep quality also shows a significant negative association [35]. A study from Finland also revealed similar results. Students with school burnout self-reported that daytime sleepiness and nighttime sleep disturbance are common in their daily lives [36]. In summary, school burnout may mediate the relationship between academic stress and sleep quality in adolescent populations.

### 2.4. The Relationships between Anxiety and School Burnout

Anxiety is a relatively common psychiatric disorder, mainly manifested as a mental and physical state characterized by cognitive, somatic, emotional, and behavioral aspects [37]. In fact, anxiety is a psychological reflection of individual external stress, threat, and anxiety. Specifically, an individual’s tendency to experience anxiety reflects timely trait anxiety, whereas state anxiety is a long-term reaction after the outcome is assessed as threatening. In addition to physiological factors, anxiety often leads to deviations or abnormalities in behavior. Previous research reported that high intensity interactions between an individual and work environment can cause anxiety, which in turn can lead to burnout [38]. Moreover, high levels of anxiety are more likely to lead to school burnout. A study of 1,868 adolescents in Slovenia showed that anxiety is significantly and positively correlated with school burnout [39]. The Anxiety Buffer Disruption Theory also suggests that the failure of buffering mechanisms can cause anxiety, resulting in abnormal behavior, such as increased burnout levels [40]. Similarly, anxiety is often positively related to school burnout for adolescents. A study of 651 students in Nepal showed that anxiety is highly prevalent in the student population and lead to more school burnout [41]. Studies from Spain also found the consistent findings. In a sample of 1021 students, during and after the Coronavirus disease 2019 (COVID-19) lockdown, as the level of anxiety increased among students about to take the exam, their level of school burnout also increased [42]. The above evidence suggests that anxiety affects adolescents’ school burnout, and thus, the indirect effect of academic stress on adolescents’ sleep quality may be chain mediated by anxiety and school burnout.

Based on the literature review, we proposed the following hypothesis:

**H1:**  *Academic stress is positively correlated with adolescents’ sleep quality.*

**H2:**  *School burnout can mediate the associations between academic stress and adolescents’ sleep quality.*

**H3:**  *Anxiety can mediate the associations between academic stress and adolescents’ sleep quality.*

**H4:**  *Adolescents’ anxiety could positively predict school burnout, and the two variables play serial mediating roles in the association between academic stress and adolescents’ sleep quality.*

## 3. Method

### 3.1. Participants and Procedures

The data was collected through a cross-sectional study carried out in Jiangsu Province. The participants were selected with the random sampling method. The procedures are as follows: firstly, we randomly chose two senior high schools and four middle schools from the school list. Secondly, two classes from each grade were randomly selected (grade 7−9 in middle schools and grade 10−12 in senior high schools). In total, 36 classes were chosen for this investigation. Thirdly, all the students in 36 classes were chosen to take part in the survey. Overall, 1309 students were invited to respond to the questionnaires. When we provided the informed consent form to the chosen youngsters and their guardians, 43 of the students or parents refused to sign it. We eventually obtained 1232 validated questionnaires after removing 34 invalid ones.

The researcher made contact with the education department and schools in advance of the investigation to establish the sample strategy. The researcher explained the goal of the study to the students before distributing the surveys. The questionnaire was given to the students who had completed the informed consent form in the classroom during their after-school activities by the research assistants, and it took around 30 min to complete. The study has received approval from the research ethics board.

### 3.2. Measures

#### 3.2.1. Academic Stress

The educational stress scale for adolescents (ESSA) was used to test the academic stress of adolescents in this study [43]. This scale has 16 items on a five-point Likert scale, with the responses from “strongly disagree to strongly agree” denoted by "1 to 5" points. A higher score suggested a stronger level of academic stress when we assessed the average score. The Cronbach’s alpha for this scale in our study was 0.909.

#### 3.2.2. Anxiety

The social anxiety scale was adopted to measure anxiety in the present study [44]. For each of the 10 items, the results ranged from "never = 1" to "always = 5". Three dimensions, including emotional, cognitive, and behavioral aspects were used to measure adolescents’ social anxiety. A higher score showed that teenagers have a stronger level of anxiety, according to our average social anxiety score calculation. The Cronbach’s alpha of the three sub-scales were 0.917, 0.881, and 0.876, respectively.

#### 3.2.3. School Burnout

In this investigation, school burnout in adolescents was assessed using the school burnout inventory (SBI) created by Salmela-Aro et al. [30]. The scale consisted of a 9-item questionnaire. A score between 1 and 6, with 6 being the highest, was assigned to each item. An example of this scale is the statement "I didn’t learn well with my homework." To determine the levels of adolescent school burnout, the scores are averaged to generate a mean score. Higher scores reflect more school burnout symptoms. In our investigation, this scale’s Cronbach’s alpha was 0.869.

#### 3.2.4. Sleep Quality

In this study, the Pittsburgh sleep quality index was utilized to evaluate adolescents’ sleep quality [45]. There were 14 items in total. Responses were collected using a four-point Likert scale, with the values "none = 0" to "three or more = 3". To determine the levels of adolescents’ sleep quality, the scores are averaged to generate a mean score. Higher scores suggested poorer sleep quality among adolescents. The Cronbach’s alpha for this scale in our investigation was 0.827.

### 3.3. Data Analysis

For data analysis, SPSS 25.0 and Amos 25.0 were used. Using SPSS, we computed the descriptive statistics for the socio-demographic factors and key variables, as well as the correlations between key variables. Structural equation modeling (SEM) with maximum likelihood estimation was used to test the model fit in the present study. The main and mediating effect tests were run with Amos 25.0. In the current study, the model fit was evaluated using the three criteria of x^2^, CFI, and RMSEA. The data appeared to fit the fictitious model, according to non-significant values of x^2^, CFI > 0.9, and RMSEA < 0.08.

## 4. Results

### 4.1. Sociodemographic Characteristics

In the sample of 1232 valid questionnaires, the descriptive statistical results are described, including, gender, age, grade, household registration, and parental education. In terms of gender, there are 528 males (42.9%) and 704 females (57.1%). The adolescents’ age varies from 12 to 18 years, with a mean age of 15.0 years and a standard deviation of 1.83. In terms of grade level, there are 742 junior high school students (60.2%) and 490 high school students (39.8%) in the sample. A total of 55.7% of the adolescents have urban household registration. In regard to their parental education, 56.5% fathers and 42.4% mothers have high school or higher education, Table 1.

### 4.2. Descriptive Statistics and Correlations among Main Variables

In SPSS 25.0, Pearson correlations are calculated (Table 2). At a significance level of 0.01 for this study, all variables are associated with one another. The findings imply that academic stress is positively connected with anxiety, school burnout, and sleep quality. Anxiety is positively correlated to school burnout and sleep quality. Moreover, school burnout and sleep quality positively correlate with one another.

### 4.3. Structural Equitation Model

**Figure 1:**
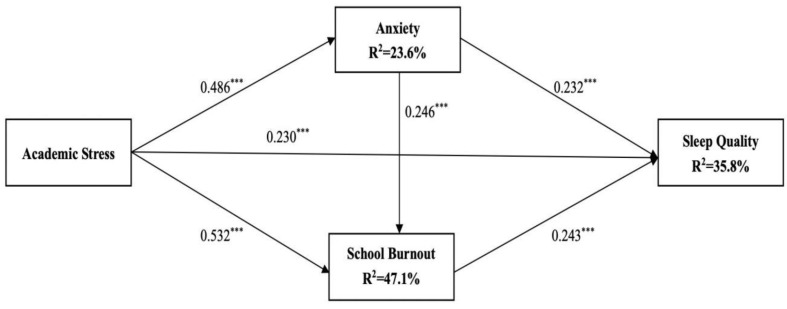
Overall structure equation model (*** *p* < 0.001).

Structural equation modeling (SEM) with maximum likelihood estimation is used to test the model fit in the present study. The results of SEM show a good model fit to the data. x^2^ = 28.984, *p* < 0.001, CFI = 0.974, RMSEA = 0.079. Figure 1 demonstrates the paths of the model.

Figure 1 shows that academic stress has a positively directly effect on adolescents’ sleep quality (β = 0.230, *p* < 0.001), suggesting that adolescents with higher levels of academic stress tend to have poor sleep quality. The results also show that the impacts of academic stress on adolescents’ sleep quality are mediated by anxiety and school burnout. Higher levels of academic stress are significantly associated with higher levels of anxiety (β = 0.486, *p* < 0.001), which, in turn, predicts poorer sleep quality (β = 0.232, *p* < 0.001). Thus, anxiety could partially mediate the association between academic stress and adolescents’ sleep quality. Moreover, higher levels of academic stress are also significantly associated with more school burnout (β = 0.532, *p* < 0.001), which, in turn, predicts poorer sleep quality (β = 0.243, *p* < 0.001). Therefore, school burnout could also partially mediate the association between academic stress and adolescents’ sleep quality. The findings also point to a direct link between anxiety and adolescents’ school burnout, with higher levels of anxiety being associated with higher levels of school burnout (β = 0.246, *p* < 0.001). Thus, anxiety and school burnout could play a chain mediating effects between academic stress and sleep quality.

The results demonstrate that only gender has a significantly effect on sleep quality. Specifically, gender has a negatively effect on adolescents’ sleep quality (β = 0.082, *p* < 0.05). These mean that females have poorer sleep quality. Together, the general model explained 35.8% of the variation in adolescents’ sleep quality, 23.6% of the variation in adolescents’ anxiety, and 47.1% of the variation in adolescents’ school burnout. The unstandardized and standardized results of the model are presented in Table 3.

## 5. Discussion

The present study reveals potential mechanisms between academic stress and sleep quality among Chinese adolescents by incorporating anxiety and school burnout as mediating factors in a comprehensive framework. The results are consistent with all hypotheses.

### 5.1. The Direct Effect of Academic Stress on Sleep Quality

Our study finds that academic stress directly contributes to poor sleep quality in Chinese adolescents, supporting Hypothesis 1. This finding is consistent with previous research confirming that adolescent academic stress can cause poor sleep quality [46]. Given the scarce resources for basic education in mainland China, academic stress—which is shaped by learning content, performance ranking, and opportunities for advancement—increases the risk of adolescents’ physical and mental health and directly affects their sleep quality [47]. Moreover, among secondary school students, as their academic stress increases, their sleep quality worsens. According to the national-scale psychiatric epidemiological survey for children and adolescents in China, their sleep disorders, sleep deprivation, and insomnia are mainly related to excessive academic stress [48]. This result also validates the stress process model, indicating its applicability in the Chinese adolescent population. This finding is also confirmed in previous literature. For example, a study conducted on adolescents in Chongqing shows that the accumulation of academic stress over time has a negative impact on sleep quality and even persists until college [49]. Therefore, academic stress should be paid more attention when dealing with adolescents’ sleep problems. The school social workers could guide teachers to rationalize their homework and help them to deal with students’ academic stress.

### 5.2. The Mediating Role of School Burnout

Moreover, our findings also support Hypothesis 2 by demonstrating the mediating role of school burnout between academic stress and sleep quality. This finding is consistent with the transactional model, which considers stress as a transactional relationship between the individual and the environment [50]. Stress can be distressing to individuals if they cannot change their ability and resources to cope with stress responses. For adolescents in mainland China, school burnout is common due to heavy academic stress, probably because they are still at the stage of physical and mental development with insufficient resilience and available external resources, resulting in psychosomatic dysfunction. At the same time, school burnout hinders their normal behaviors and leads to a decrease in their sleep quality. This conclusion has been confirmed by previous empirical studies. For example, high academic stress can exceed adolescents’ ability to cope with stress and lead them into negative aversion to school, which directly affects their sleep problems [51]. In fact, academic stress among Chinese adolescents has received widespread attention. By contrast, school burnout caused by academic stress has been neglected in research and practice. Due to the mediating effect of school burnout on the relationship between academic stress and sleep quality, the results of this study provide new evidence to support the intervention of clinical psychologists and school social workers. The findings can guide them to distinguish the different manifestations of stress and school burnout, and help students to cope with stress and school burnout in proper ways.

### 5.3. The Mediating Role of Anxiety

The findings also support Hypothesis 3, suggesting the mediating role of anxiety on the association between academic stress and sleep quality. This result suggests that in the educational context of mainland China, adolescents’ anxiety originates from academic stress caused by heavy school tasks, and hence reduces their sleep quality. Furthermore, anxiety plays a mediating role in the association between academic stress and sleep quality. Despite the implementation of the policy of “reducing the burden of schoolwork” in mainland China, adolescents’ academic stress remains heavy, given the competition for higher education and grade ranking [52]. This scenario greatly impairs their anxiety-buffering mechanisms and leads to their anxiety problems. This result also verifies the applicability of the stress process model on Chinese adolescents, indicating that academic stress is indeed an important risk factor for their anxiety [9]. Moreover, Chinese adolescents’ sports and leisure activities are compressed, and all their time is spent on attending academic tutorials. This further exacerbates their level of anxiety, resulting in problems with their sleep quality.

### 5.4. The Chain Mediation Role of Anxiety and School Burnout

In addition, we found a significant effect of anxiety on school burnout, confirming a chain mediating effect on the relationship between academic stress and adolescents’ sleep quality. Thus, Hypothesis 4 is also verified. For adolescents, psychological health is the most important guarantee to be able to live and study normally. However, in mainland China, the educational environment causes adolescents’ time and energy to be consumed in their studies. In addition to academic stress, increased levels of individual anxiety also lead to school burnout, illustrating that as a psychological and physiological abnormal state, anxiety often leads to sleep quality problems such as sleep deprivation and inefficiency. Moreover, this result verifies previous studies, such as that of Turnipseed, which concludes that anxiety leads to burnout [38] and that people with high levels of anxiety have a high likelihood to have burnout. Therefore, from this perspective, to solve the problem of school burnout among Chinese adolescents, we should not only focus on reducing academic stress but also pay attention to their anxiety levels.

Although the effects of academic stress on adolescent sleep quality have been studied previously, most of the current literature has focused on social support [53], Internet addiction [54], and physical activity [55]. In fact, the effects of anxiety and school burnout on adolescents’ sleep quality are more severe in the educational context of mainland China [56], but their chain mediating effect has been less studied. Therefore, this study deepens our understanding by verifying the chain mediating effect of anxiety and school burnout on the influence of academic stress on adolescents’ sleep quality, thus promoting the further development of research on the relationship between academic stress and sleep quality and providing empirical evidence for the intervention of adolescents’ related problems.

## 6. Implications

### 6.1. Theoretical Implications

This study has some theoretical implications. Firstly, this study innovatively explored the process of academic stress affecting adolescents’ sleep quality. Although previous studies have revealed the associations between academic stress and sleep quality [18], the present study is one of the rare studies that simultaneously analyzed academic stress, internalization of psychiatric symptoms, and externalization of negative behaviors in one study. Secondly, this study investigated the chain mediating effects of anxiety and school burnout on the associations between academic stress and adolescents’ sleep quality. Moreover, the present study corroborates previous research from a physiological perspective that stress induces untimely excitability in certain areas of the cerebral cortex, thereby decreasing sleep quality [57]. In addition, the empirical evidences of this study also validate the transactional model and the stress process model, suggesting that these theories also have applicability in the Chinese context.

### 6.2. Practical Implications

This study also has several practical implications. Since academic stress is an important risk factor causing poor sleep quality in adolescents, future practical directions should be centered on reducing the academic burden of adolescents. For example, we should enhance the efficiency of classroom learning, eliminate the off-campus training, and discard the grade ranking among students, so as to free them from heavy academic tasks [58]. At the same time, students’ time for extracurricular activities should be increased, including encouraging participation in sports, cultivating their hobbies, getting close to nature, reading extracurricular books, developing peer friendships, etc. [59]. It is worth noting that the implementation of these approaches should not only target the adolescents, but also include stakeholders such as families, schools, and policy makers to work together to alleviate adolescents’ academic stress, thus alleviating the problems of poor sleep quality.

Furthermore, this study suggest that anxiety and school burnout can mediate the relationship between academic stress and sleep quality. In order to solve the academic stress and sleep problems, we suggest that parents and teachers should increase their awareness of adolescents’ anxiety and school burnout. At the same time, psychological counselors should also improve their skills in identifying school burnout and anxiety to accurately grasp adolescents’ negative emotions and abnormal psychiatric symptoms. Moreover, social workers should help adolescents to self-regulate their emotions and help them to improve their resilience so as to adjust and release their negative emotions [60]. In addition, educators should provide adolescents with psychological counseling and intervention so that they can relieve their negative emotions and psychiatric symptoms caused by stress in a timely manner and ultimately achieve the goal of solving maladaptive behavior problems.

## 7. Limitations

This study still retains several limitations. First, this study uses a self-report method, and the respondents’ condition may be influenced by social desirability bias and the COVID-19 lockdown. Second, the results of the effect of academic stress on adolescent sleep quality may also be influenced by other factors, such as school climate and peer relationships, which can be considered in the future. Third, given that this study was carried out with cross-sectional data, we cannot reveal the causal relationship between the factors. Moreover, the sample data were collected across a small range and cannot be generalized to a larger sample. Thus, the generalizability of the results of this study merits further validation.

## 8. Conclusions

This study investigates the associations among academic stress, anxiety, school burnout, and sleep quality among Chinese adolescents. The findings demonstrated that academic stress can directly and indirectly affect adolescents’ sleep quality. Moreover, a chain mediating effect of anxiety and school burnout occurs between academic stress and sleep quality. This study reveals the influencing mechanism between academic stress and adolescents’ sleep quality. In addition, the findings may shed light on social policy and social work intervention toward adolescents’ sleep quality.

## Figures and Tables

**Table 1:** Sociodemographic characteristics (*N* = 1232).

	Frequency *(N)*	Percentage (%)
Gender		
Male	528	42.9
Female	704	57.1
Age (Range 12−18)	Mean = 15.0	S.D. = 1.83
Grade		
Grade 7	324	26.3
Grade 8	180	14.6
Grade 9	238	19.3
Grade 10	130	10.6
Grade 11	164	13.3
Grade 12	196	15.9
Household registration		
Rural	546	44.3
Urban	686	55.7
Father’s education		
Illiterate	24	1.9
Primary school	78	6.3
Middle school	434	35.2
High school	358	29.1
Vocational school	150	12.2
Junior college	110	8.9
University or higher	78	6.3
Mother’s education		
Illiterate	86	7.0
Primary school	160	13.0
Middle school	464	37.7
High school	256	20.8
Vocational school	118	9.6
Junior college	92	7.5
University or higher	56	4.5

**Table 2:** The results of Descriptive statistics and Correlations among main variables.

	Mean	S.D.	1	2	3	4
1. Academic stress	3.33	0.81	1			
2. Anxiety	2.76	1.01	0.486 **	1		
3. School burnout	2.86	1.10	0.652 **	0.505 **	1	
4. Sleep quality	1.73	0.55	0.503 **	0.479 **	0.511 **	1

** *p* < 0.01.

**Table 3:** The unstandardized and standardized estimates of the SEM model.

Predictors	Outcomes	B	β	S.E.	C.R.	*p*
Academic stress	Sleep quality	0.156	0.230	0.031	5.117	***
Academic stress	Anxiety	0.605	0.486	0.044	13.800	***
Academic stress	School burnout	0.725	0.532	0.046	15.866	***
Anxiety	Sleep quality	0.127	0.232	0.021	6.029	***
School burnout	Sleep quality	0.122	0.243	0.022	5.477	***
Anxiety	School burnout	0.269	0.246	0.037	7.318	***
Gender	Sleep quality	0.091	0.082	0.036	2.522	*
Grade	Sleep quality	0.009	0.031	0.010	0.916	0.360
Household registration	Sleep quality	0.014	0.012	0.036	0.378	0.705

*** *p* < 0.001; * *p* < 0.05

## Data Availability

The data presented in this study are available on request from the corresponding author.
